# Radioprotective and Radiomitigative Effects of Resveratrol in Radiation-Induced Reproductive Toxicity in Male Mice

**DOI:** 10.3390/toxics13121019

**Published:** 2025-11-26

**Authors:** Małgorzata M. Dobrzyńska, Aneta Gajowik

**Affiliations:** Department of Radiation Hygiene and Radiobiology, National Institute of Public Health NIH–National Research Institute, 24 Chocimska Street, 00-791 Warsaw, Poland; agajowik@pzh.gov.pl

**Keywords:** irradiation, resveratrol, sperm count, sperm quality, recovery, DNA damage, sperm morphology

## Abstract

**Background**: Agents with free radical-scavenging functions may act as radiation modifiers, protectors, or mitigators. **Methods**: We investigated whether supplementation with resveratrol (RSV) in mice, at different times after the beginning of X-irradiation, may influence sperm count and quality during the irradiation and recovery. **Results**: Irradiation importantly decreased the sperm count. RSV supplemented with 1 Gy since 24 h increased sperm count. The combination of low doses increased, whereas the combination of high doses reduced DNA damage. Coadministration of two high doses since the eighth day significantly increased DNA damage and slightly increased sperm count. The supplementation of RSV during recovery was toxic to irradiated males. The sperm parameters were a little better in the absence of RSV. The degree of DNA injury of germ cells was importantly lower in groups combined with 1 Gy. **Conclusions**: Resveratrol counteracted the radiation-induced death of germ cells and improved the sperm count. RSV may function as radioprotector (before or during exposure) and radiomitigator (after exposure) of lethal effects in male gametes. The combination of high doses of irradiation with RSV over 24 h mitigated DNA damage. Contrarily, supplementation during recovery is not recommended since it may be toxic during long-lasting irradiation.

## 1. Introduction

Human jobs, including the use of radiation and radioactive materials, are sources of human-made radiation subjection, together with natural exposure from cosmic rays and naturally occurring radioactive materials. The use of radioactive materials in production, cultivation and science is expanding, increasing the risk of adverse effects from mishandled sources [1]. Moreover, it is known that 60% of cancer patients received radiotherapy, so it is interesting and useful to identify possible protectors of normal tissues that are inevitably or accidentally exposed to ionizing radiation during such procedures [2].

Spermatogenesis is a particularly important process, which takes place within seminiferous tubules of the testes. This process results in the maturation of the male germ cells from spermatogonial stem cells through spermatocytes and spermatids to spermatozoa, ready to fertilize eggs. Previous studies showed that genetic damage induced by radiation or chemicals might be passed on to the progeny causing male-mediated developmental toxicity [3]. Both males and females donate half of the genetic material to the genome of evolving children, so the transmission of congenital abnormality via the sperm is incredibly noteworthy [4].

Ionizing radiation is a well-known mutagenic and carcinogenic factor. Radioactive isotopes attend in soil, water and air, and cosmic rays classified as natural sources. However, the greatest origin of human subjection is considered to be diagnostic radiology and radiation therapy used for health care. The global mean background subjection for a human is determined to be about 2.4 mSv per year, but then the mean from medical diagnosis varied from 0.043 to 2 mSv per year [1,5]. Ionizing radiation has an adverse effect on male gametes. The most radiosensitive organ in the body is the testes together with germ cells [6]. Correspondingly, the International Commission on Radiological Protection [7] report the weighting factor for the gonads is 0.08. In employees’ job-related subjection to ionising radiation, several authors observed changes, such as diminished quantity, motility, elevated sperm malformations, sperm fragmentation and global hypermethylation in humans [8,9,10,11].

Resveratrol (trans-3,4,5-trihydroxystilbene, RSV) is a natural, non-flavonoid polyphenol from a group of stilbenes, the compounds, which characterize significant biological activities. It is structurally similar to diethylstilbestrol and estradiol, and due to this it is called phytoestrogen. RSV is present in fruits, such as grapes, peanuts, strawberry, blueberry, cranberry, mulberry, lingberry, sparkleberry, bilberry, and in wine, especially in red wine [12,13]. Its nutritional ingestion is approximately 100 µg daily [14]. RSV is also generated by various plants in response to injury, stress, bacteria and fungi infections, UV radiation and exposure to ozone [15,16]. RSV shows many pro-health attributes, such as antioxidant, anticarcinogenic, anti-inflammatory, neuroprotective, antidiabetic, analgesic, antiviral, and cardioprotective action [17,18,19,20,21,22,23]. RSV is widely present in the Mediterranean diet, which is known for its beneficial properties for health, mainly for prevention of cardiovascular diseases despite a high fat diet [24,25].

RSV is also considered as a modulator or protector of ionizing radiation-induced damage [26,27,28,29]. Contrary, there are also reports that showed that RSV intensifies the radiosensitivity of cancer cells [30,31,32].

The study aimed to evaluate how the timing of RSV supplementations influences sperm count and quality during irradiation and recovery.

## 2. Materials and Methods

### 2.1. Animals and Exposure

Seven-week-old male Swiss outbreed laboratory mice obtained from the “Kołacz” Animal Breeding Laboratory (Warsaw, Poland), were housed in standard rodent cages in a room with controlled temperature, humidity, and light cycles (12 h dark, 12 h light). Tap water and rodent diet were available *ad libitum*. The mice were randomly assigned to either the control or exposed groups, one week after acclimatization. Eight-week-old male mice were exposed to RSV dissolved in a small amount of ethanol and diluted in drinking water to obtain the desired dose (7 mg/kg body weight (bw) or 28 mg/kg bw, daily), irradiated with X-rays (0.5 Gy or 1 Gy daily), or exposed to a combination of both agents (0.5 Gy + 7 mg/kg bw RSV, 0.5 Gy + 28 mg/kg bw RSV, 1 Gy + 7 mg/kg bw RSV, 1 Gy + 28 mg/kg bw RSV daily). Animals were irradiated 5 times per week (working days), i.e., 10 times during the whole experiment, whereas RSV was continuously supplied in drinking water starting from 24 h or 1 week following the initiation of daily irradiation. Body weight of the animals was checked weekly, and the volumes of control water and RSV–water solution were checked daily. The solution of RSV in water was prepared twice a week. A therapeutic Roentgen unit Medicor type THX-250 (Budapest, Hungary) was used for irradiation, and operated with the following parameters: 155 kV, 18 mA, added filtration 0.25 mm Cu and HVL 2 mm Al. The unit was used previously for medical purposed in human investigation. Mice were subjected to whole-body irradiation at the dose rate of 0.20 Gy/min. For irradiation each mouse was placed in a plastic tube. The tubes with the mice were placed on a circular base. The mice’s heads were closest to the center of the circle. Up to 5 mice were exposed to the same dose at the same time. The total doses were 5 Gy or 10 Gy during 2 weeks. The above doses approximately correspond to those allowed for individuals occupationally exposed to radiation. According to the recommendation of the International Commission on Radiological Protection from 2007, the effective dose limit for occupational exposure is 20 mSv/year. Over the next five calendar years its cumulative value doses must not exceed 100 mSv [7]. Radiosensitivity is both individual and species = specific. A factor of 10 is usually applied to extrapolate the response of an animal model to that of average human, i.e., a human would be 10 times more sensitive than the most sensitive animal model. Another coefficient of 10, referring to individual variability in the population [33] was also taken into consideration. Thus:

50 mSv × 10 × 10 = 5000 mSv = 5 Sv,

100 mSv × 10 × 10 = 10,000 mSv = 10 Sv.

For X-rays and gamma radiation, 1 Sv = 1 Gy. Therefore, the selected total doses for animal studies were 5 and 10 Gy, which corresponded to effective doses 50 and 100 mSv, respectively, resulting from occupational exposure in humans.

Control animals were sham irradiated and unexposed to RSV, or were exposed only to RSV. The groups of animals were sacrificed 24 h after the last irradiation. Other groups of animals were observed during recovery process for additional 2-weeks. During the recovery there were two different groups of mice, with RSV (recovery in the presence of RSV–RPR) or without RSV (recovery in the absence of RSV–RAR).

Eight to ten mice were used for each of the doses and time periods.

### 2.2. Reagents

Resveratrol HPLC (*trans*-Resveratrol) originated from Carl ROTH GmbHKarlsruhe, Germany, distributor Linegal Chemicals (Warsaw, Poland), catalog no.: N811.1; purity ≥ 98%; M = 228.25 g/mol.

Ethyl alcohol 70% originated from POCH Company, Gliwice, Poland. RPMI 1640 Roswell Park Memorial Institute Medium with glutamine was bought from Life Technologies (Thermo Fisher Scientific, Waltham, MA, USA); Gibco ^TM^, Carlsbad, CA, USA. Other reagents were supplied from Sigma-Aldrich, St. Louis, MO, USA.

### 2.3. Sperm Count and Quality

Both testes and epididymides were removed and weighed from each male. One epididymidis was macerated in 0.2 mL of 1% solution of trisodium citrate for 5–8 min and minced. Then the solution was made up to 2 mL and mixed for about 1 min. The sperm suspension was diluted 1:1 in 10% buffered formalin. The spermatozoa were counted using an improved Neubauer hemocytometer [34,35].

The content of the second epididymides were placed into 0.2 mL of warm (37 °C) physiological saline. An aliquot was placed on warm (37 °C) microscope slide and covered with a cover slip. Two hundred cells per animal were evaluated for motility within 5 min after killing the animal [36].

The remaining sperm was distributed evenly in the saline. The study of frequency of morphologically abnormal spermatozoa was performed according to the procedure described by Wyrobek and Bruce [37]. Smears were prepared on microscope slides, air-dried overnight and stained with eosin Y. Then, one thousand spermatozoa per mouse were analyzed using a light microscope, and abnormal spermatozoa morphology (e.g., lacking hook, amorphous, banana-shaped head, two tails, head folded on itself, two heads) were recorded.

### 2.4. Comet Assay

For the Comet assay the method of tissue preparation described previously was used [38]. One testis from each animal was decapsulated and placed in the RMPI 1640 medium and minced with scissors. Before using the cells, tubes were swirled so that single cells remained in the suspension. 5 μL of cell suspension were mixed in an Eppendorf tube with 75 μL low melting point agarose (LMA) for embedding on slides. The slides were immersed in alkaline lysis (2.5 m sodium chloride (NaCl), 100 mM ethylene diamine tetra-acetic acid, sodium salt Na_2_ (EDTA), 10 mM Tris, 1% sodium sarcosinate, pH 10) overnight at 4 °C. Then, they were drained and placed in gel electrophoresis tank, and left in the solution for 20 min. The electrophoresis was conducted at 4 °C for 20 min using 19 V and 300 mA. Afterwards, neutralization slides were stained with EtBr and examined using a fluorescence microscope (Nikon, Tokyo, Japan). Images of one hundred randomly selected cells from each animal were recorded and analyzed using CASP image-analysis software [39]. The Comet tail moment and % DNA in the Comet tail was chosen to determine induction of DNA breaks.

### 2.5. Statistical Analysis

A commercially available program Statistica v.9 (StatSoft, Kraków, Poland), was used to perform the statistical analysis. After determining that the measured variables had a probability distribution similar to the normal distribution, differences between groups were compared by one-way analysis of variance (ANOVA), which allows for the comparison of the significance of differences between the means of more than two groups of studied variables. The significance level was established at *p* < 0.05. For significant interactions, a post hoc Fisher’s (Lowest Significant differences) test was then performed.

## 3. Results

**Figure 1:**
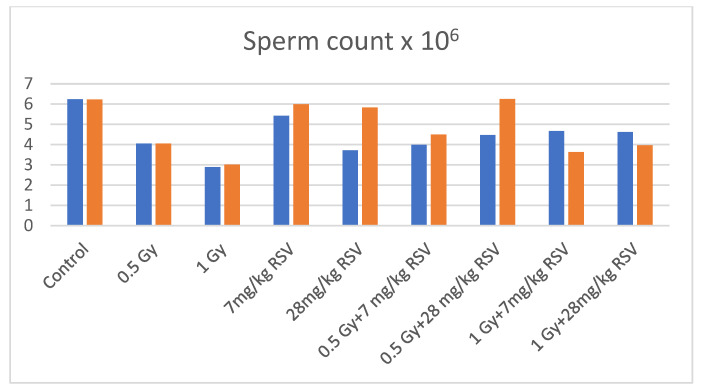
Sperm count of mice supplemented with resveratrol since 24 h (blue bars) or 8th day (red bars) after the start of irradiation.

The results of irradiation mice supplemented with RSV since 24 h after the first exposure to X-rays are shown in Table 1. The body weights of males significantly decreased in all groups except of 28 mg/kg bw of RSV and 0.5 Gy compared to control. In groups 0.5 Gy + 7 mg/kg bw RSV and 0.5 Gy + 28 mg/kg bw of RSV the body weight was also reduced as compared to 0.5 Gy alone. The testes were not pathologically changed. The average testes weight was importantly reduced in all irradiated and combined groups as compared to control and additionally in groups 1 Gy + 7 mg/kg bw RSV and 1 Gy + 28 mg/kg bw of RSV as compared to 1 Gy. The mean epididymis weight was reduced in irradiated groups and in combined groups except of 0.5 Gy + 7 mg/kg bw of RSV and 0.5 Gy + 28 mg/kg bw of RSV compared to the control and was increased in both mentioned groups as compared to 0.5 Gy alone. The average sperm count was significantly decreased after irradiation with 0.5 Gy or 1 Gy daily as compared to the control and increased in combined with 1 Gy groups as compared to irradiation alone [Figure 1]. The sperm motility was decreased in all groups except of 7 mg/kg bw of RSV. The percentage of abnormal spermatozoa and the percentage of DNA in the Comet Tail were significantly increased in the group of 0.5 Gy + 7 mg/kg bw RSV. DNA damage in this group was significantly higher as compared to 0.05 Gy alone, and lower in 1 Gy + 28 mg/kg bw group as compared to 1 Gy.

The results after supplementation of RSV since the eighth day following the beginning of irradiation are presented in Table 2. The mean body weight was importantly reduced after irradiation with 1 Gy and in combined groups, except of 0.5 Gy + 7 mg/kg bw RSV. The mean testis weight was reduced in all groups except of 7 mg/kg bw and 28 mg/kg bw RSV groups. The mean epididymes weight significantly decreased in both irradiated groups and in the group of 0.5 Gy + 28 mg/kg bw RSV compared to the control and increased in the group of 1 Gy + 7 mg/kg bw RSV compared to 1 Gy alone. The sperm count significantly reduced after irradiation to 0.5 Gy or to 1 Gy daily and in combined with 1 Gy groups. In the group of 0.5 Gy + 28 mg/kg bw RSV the sperm count was markedly increased compared to irradiation alone [Figure 1]. The motilities of spermatozoa were diminished in all experimental groups, except of 28 mg/kg bw RSV. No effects were observed on the sperm abnormality nor on the DNA damage of male gametes.

The effects of 2-weeks recovery after supplementation of RSV since 24 h following the beginning of irradiation are shown in Table 3. In groups of 1 Gy + 7 mg/kg bw RSV and 1 Gy + 28 mg/kg bw RSV almost all (7 of 9 i.e., 78% and 6 of 8 i.e., 75%) animals have died during the recovery period, whereas in the group of 1 Gy alone, only 1 male of 9 (i.e., 11%) has died. In other groups 100% of animals survived. The mean animal weights were significantly lower compared to the controls in all combined groups. Additionally, results of all combined groups except of 1 Gy + 28 mg/kg RSV (RAR) were significantly different compared to appropriate dose of irradiation alone. The mean testes weights were significantly decreased in 0.5 Gy and in combined groups as compared to the control, and in combined with 0.5 Gy RPR groups also as compared to 0.5 Gy separately. Epididymis weight was significantly higher in 1 Gy and in 0.5 Gy + 28 mg/kg bw RSV (RAR) groups. Sperm count was significantly reduced in irradiated, and in combined with 1 Gy RAR groups and in combined with 0.5 Gy RPR groups. Percent of motile spermatozoa was importantly dissimilar compared to the control in 1 Gy group. The percent of morphologically injured spermatozoa was elevated in 0.5 Gy + 7 mg/kg bw RSV (RPR), and in both combined with 1 Gy (RAR) groups compared to control and in 0.5 Gy + 28 mg/kg bw RSV RPR group compared to 0.5 Gy alone, and in 1 Gy + 28 mg/kg bw RSV RAR as compared to 1 Gy alone. There were no important changes in the degree of DNA injury.

The effects of 2-weeks recovery after supplementation of RSV since the eighth day after the beginning of irradiation are presented in Table 4. Almost all (8 of 10) males from 1 Gy + 7 mg/kg bw RSV RPR did not survive the recovery time, whereas among males exposed to 1 Gy only 2 of 10 have died. The mean body weight was significantly different in all combined RPR and RAR groups. Mean testes weight was significantly decreased in irradiated and in all combined RPR and in 0.5 Gy + 7 mg/kg bw RSV (RAR) groups compared to the control and additionally, all RPR groups and 0.5 Gy + 7 mg/kg bw RSV (RAR) group were significantly different compared to irradiation separately. The epididymes weights significantly increased in all combined RAR groups except of 0.5 Gy + 7 mg/kg RSV. This parameter was also significantly lower in the group of 1 Gy + 28 mg/kg bw RSV RPR group as compared to 1 Gy alone. Sperm count significantly decreased after exposure to 0.5 Gy and in all combined groups. Additionally results of 0.5 Gy + 28 mg/kg bw RSV RAR, 1 Gy + 28 mg/kg bw RSV (RPR), 1 Gy + 7 mg/kg bw RSV (RPR) and in 1 Gy + 28 mg/kg bw RSV (RPR) importantly differs as compared to irradiation. Percent of motile spermatozoa was not significantly different from control, except of 1 Gy + 7 mg/kg bw RSV (RAR). Additionally, the result of 1 Gy + 28 mg/kg bw RAR groups was significantly different from 1 Gy. Percentage of abnormal spermatozoa was importantly elevated in 1 Gy and all combined groups compared to the control. Additionally, the result of 1 Gy + 28 mg/kg bw RSV (RPR) was significantly higher compared to 1 Gy alone. The frequency of DNA damage of gametes was significantly lower in groups combined with 1 Gy (RAR).

## 4. Discussion

Approximately 15% of couples after 12 months of systematic unsecured cohabitation cannot become pregnant and for at least 50% of infertility occurrences the responsibility of the male component is considered [40]. The World Health Organization (WHO) characterized the male factor infertility as an alteration in sperm concentration and/or motility and/or morphology in at least one sample of two sperm analyses between 1 and 4 weeks apart [41]. The most important seems to be oligozoospermia i.e., low sperm count and quality, which is responsible for 90% of male infertility [42,43].

Male infertility is caused mainly by oxidative stress. The fertilization capacities of spermatozoa relate to the presence of controlled level of free radicals; however, the excess of oxidants or reactive oxygen species in comparison with the antioxidant defense mechanism not delivered, may be harmful for germ cells [44]. Cell membrane of spermatozoa is rich in polyunsaturated fatty acids, which consist of unconjugated double bonds containing electrons, which may be donated to reactive oxygen species (ROS), leading to the generation of lipid peroxides. As a result, the germ cell membrane is disrupted and consequently, the sperm viability and motility is decreased [45,46,47]. The decreased sperm viability takes place after the modification of membrane proteins and an atypical acrosome reaction that results in a reduction in the ability spermatozoa to fertilize oocytes. The cause of diminished sperm motility is a reduction in axonemal protein phosphorylation. ROS may also cause DNA injury in spermatozoa by a direct attack on the bases or the phosphodiester backbones. In turn, DNA damage may lead to mistaken fecundation, decreased implantation, and defective embryo development, and may consequently lead to induction of enlarged mortality and increased likelihood of developing cancer. The next consequence may be apoptosis of highly damaged gametes leading to reduced sperm count [45,46,47,48,49,50]. Sperm morphology is a result of complex cellular modifications occurring during spermatogenesis [51]. Sperm abnormalities induced by elevated ROS amount, may cause teratozoospermia and decreased fertility or temporary or permanent infertility [52,53].

Agents that show free radical-scavenging features may act as radiation modifiers or protectors, if added before or shortly after irradiation, or mitigators, which work if administered after radiation [54].

This study does not investigate radiation adaptive response (RAR). RAR is indicated by a decrease in radiobiological response in cells that have been pretreated with a low-dose radiation before a higher dose. In contrast, radioprotection occurs when the agent is administered just before or during irradiation, while radiomitigation occurs when it is applied after exposure to irradiation. RAR and pharmacological mitigation have similar biological pathways to oxidative stress regulation, DNA repair, and cellular signaling dynamics [55]. For DNA repair, DNA-dependent protein kinase (DNA-PK) and ERCC5 (XPG) may be engaged [56,57]. In cell cycle regulation and DNA repair participating ataxia telangiectasia and p53 tumor suppressor proteins are involved [56,58,59]. Adaptive response to low-dose of irradiation may require DSB repair by non-homologous end joining repair pathways. Several protein kinases are reported to activate p53, for example low-dose irradiation regulation actions p38MAPK. Contrarily, it is downregulated at high doses [60].

The primary pathway for the adaptive response to oxidative stress is the Nrf2 pathway. It is an adaptive response to environmental and endogenous stresses. Mutated (ATM) and p53 tumor suppressor proteins are connected with cell cycle regulation and DNA repair [56,58,59]. Nrf2 belongs to basic leucine transcription factors, which bind to the antioxidant responsive element (ARE) and are important for maintaining cellular redox balance. Keap1 sequesters Nrf2 in the cytoplasm, where Nrf2 then simplifies the degradation of Keap1 via the proteasome [61]. The Nrf2-antioxidant pathway may be prompted by irradiation or chemicals leading to a decrease in irradiation-induced DNA DSBs [62,63]. Upregulation of the Nrf2 pathway following low-dose irradiation may also be caused by activation of autophagy [55]. The lack of autophagy induces inhibition of Nrf2-Keap1 binding by p62 and stabilizes Nrf2 and its pathway [64]. Autophagy may also be upregulated Nrf2 by its autophagosomal degradation of Keap1 or by higher expression of the oncogene signaling protein and the following initiation of the antioxidant pathway [65,66].

Plants are naturally protected against radiation. For example, phytophenols present in many plants are structurally adjusted for the purpose of activation by electron donating substituents, which hold back energy transfer mechanisms, supressing oxidative stress and stabilising redox processing cells [67].

RSV activates antioxidant enzymes, i.e., catalase and superoxide dismutase, which are involved in lipid damage of spermatozoa [68]. This compound is able to activate anti- and proapoptotic mediators and in this way protects cells from DNA injury and apoptosis [19]. Moreover, it induces cell cycle arrest, apoptosis, differentiation and inhibits cancer cell proliferation [69].

RSV is well tolerated, harmless, does not affect the reproductive ability of both sexes and is also non-toxic in the embryo of rodents [70]. It is a relatively safe and natural medication that is rapidly absorbed, metabolized, transported and distributed; however, long-term administration is unknown [71].

Due to its abilities, resveratrol is considered to be an encouraging factor for the therapy of male infertility. RSV, although structurally like oestrogens, does not show estrogenic properties and does not affect reproductive organs [72,73].

Enlargement in the relative weights of the testes and epididymides and usual characteristics of testis, as well as increased epididymal sperm motility and testicular sperm count were observed in ICR mice given 50 mg/kg of resveratrol daily for 28 days [74]. In turn, contrasting studies show that RSV may lead to a decrease in testicular weights, disruption of seminiferous tubules morphology and spermatogenesis [75,76,77]. Other authors showed that RSV increased the relative testes weight, improved apoptotic index and testosterone levels in mice [78]. In the current study, the average body, testes and epididymis masses, as well as sperm count and quality, were not significantly different in RSV exposed groups compared to the control.

It is postulated that RSV regulates the oestrogen-response system, acting as a regulator of male reproductive function [73]. Moreover, it has capacity to pass through the blood–testis barrier, conveying its protective influence in the testis [79]. RSV directly stimulates the hypothalamic–pituitary–gonadal axis, with no disagreeable influences on testes and promotes spermatogenesis by ameliorating the effect induced by 2,5-hexanedione [73].

In humans, RSV caused enhancement of sperm concentration and count [80], and decreases in DNA damage in fertile and infertile patients [81,82,83,84]. RSV also caused sperm production and motility in laboratory animals, as well as enhanced blood testosterone levels, testicular sperm count and sperm motility in rabbits [74]. Lower doses of RSV counteract lipid peroxidation, protecting sperm chromatin and plasma membranes [73]. It is a strong blocker of the oxidation of polyunsaturated fatty acids present in lipoprotein [85]. Hence, RSV could be operative by reducing the frequency of ROS and proinflammatory components in seminiferous tubules, thus enhancing sperm and androgen construction [73]. As earlier papers showed, RSV at low doses ameliorates cell viability, but at high doses is cytotoxic [86,87]. This may be useful in cancer therapy. Moreover, RSV is able to preserve sperm chromatin texture, but not acrosome [83]. Low concentration has a favorable effect on the sperm motility, but higher concentrations showed opposite results [86,88,89,90,91].

RSV can mitigate oxidative stress through several processes, such as reduction in the generation of ROS, directly scavenging free radicals, increasing the level of endogenous antioxidant enzymes, stimulation of antioxidant particles and the expression of connected genes involved in mitochondrial energy biogenesis, predominantly through AMPK/SIRT1/Nrf2, ERK/p38, mitogen-activated protein kinase (MAPK), and PTEN/Akt signaling pathways, and inducing autophagy via mTOR-dependent or TFEB-dependent pathway [92]. RSV may successfully remove free radicals and increases the activities of SOD, CAT, and GPX [93,94]. Cytoprotective effects of RSV are mainly caused by mitigation of mitochondrial ROS [95]. The hormetic characters of RSV can be imputed to its dose-dependent dual acting influence on cellular redox state, i.e., an antioxidant at low concentration and a pro-oxidant at high doses [96].

According to our knowledge, there are no issues regarding the influences of RSV on irradiated male germ cells. The current study reported effects on the germ cells in irradiated male mice co-administered with RSV since different times after the beginning of irradiation and compared the effects during convalescence in the presence or absence of RSV.

The 2 weeks of irradiation significantly diminished the sperm count and quality similarly to earlier results [97,98,99,100]. The current study showed that irradiation leads to a death of approximately 35 to 54% of germ cells and confirmed that ionizing radiation diminishes sperm count and quality. The reduced testes and epididymis weights correlated with the diminished sperm count. The diminished sperm count after irradiation is mainly assigned to apoptosis of germ cells with genomic abnormalities started probably by Tp53 protein [8].

In the mice, the spermatogenesis cycle continues for 8 weeks, so during 2 weeks of the experiment all stages of male germ cells present in the animal body were irradiated simultaneously. After 2 weeks of exposure the results of irradiation on the spermatozoa and late spermatids were shown, whereas after an additional 2 weeks of convalescence on the early spermatids and late spermatocytes, the doses used did not induce sublethal nor chronic testicular damage. They affected only exposed germ cells.

As earlier papers showed, the male reproductive organs and cells are susceptible to ionizing radiation. Spermatogonial stem cells and differentiating spermatogonia are highly sensitive. Spermatocytes are less sensitive, while spermatids are rather radioresistant [101,102,103]. The current study did not confirm the above finding, since spermatids, spermatocytes and spermatozoa showed high sensitivity to irradiation.

Our study showed that co-administration of RSV since 24 h following the beginning of 1 Gy irradiation daily importantly enhanced the sperm count and additionally, the combination with a high dose of RSV reduced the frequency of DNA damage. Contrarily, the combination of 7 mg/kg bw RSV with 0.5 Gy of irradiation leads to increased DNA damage and increased percentage of abnormal spermatozoa.

In relation to coadministration of 1 Gy + 28 mg/kg of RSV since the eighth day after the first irradiation, the significant increase in DNA damage and slight increase in sperm count was noted. In turn, the sperm count was markedly improved following combined exposure to 0.5 Gy of irradiation and RSV. This correlated with an increase in the testes weight. The best result was seen after exposure to 0.5 Gy + 28 mg/kg bw RSV, where the level of sperm count reached the control value. The above results confirmed the earlier results of RSV alone, i.e., increased the level of sperm count and concentration [73,80,88,89]. Reduced DNA damage may indicate stimulation of DNA repair–DNA damage-control biosystem by low dose radiation [104]. RSV reduces germ cells apoptosis in rodents and protects the male reproductive tract [105,106]. According to results of other studies, RSV increases sperm creation in rats by stimulating the hypothalamic–pituitary–gonadal axis in absence of harmful results [73].

Corresponding with our outcomes, in the paper of Juan et al. [73], sperm count was importantly (about 1.7 times) increased in the resveratrol-treated male rats collated to the control group; nevertheless, the sperm quality did not vary. The expansion in the sperm creation noted as a result of RSV administrationmay also be generated by a general enlargement in the size of spermatogenic tissue (reduction in the average diameter of the seminiferous tubules with enlarge in the testicular tubules density) observed when RSV is co-administered with isoflurane [107]. The current results for germ cells confirmed our earlier results for somatic cells, where RSV reduced the frequency of micronuclei induced by irradiation in reticulocytes [28].

Results of convalescence showed that longer exposure to RSV (4 weeks) is harmful to male mice irradiated before. In the experiment regarding mice supplemented with RSV since 24 h after the first irradiation, almost all males have died when RSV was supplemented for an additional 2 weeks, whereas after irradiation to 1 Gy alone only one has died. Surpassingly, the germ cells parameters were better after the recovery of the absence of RSV. In the occurrence of supplementation of RSV since the eighth day after the first irradiation (3 weeks of RSV exposure), the majority of males exposed to 1 Gy + 7 mg/kg bw RSV have died. This may confirm earlier results regarding to toxic effects of RSV after longer administration i.e., after higher doses [86,87].

Results after convalescence i.e., when cells were subjected as late spermatids and early spermatocytes, showed that RSV at each dose given within 2 weeks after the end of irradiation decreased the sperm count and increased the percent of abnormal spermatozoa collated to the results of 0.5 Gy alone. The response was slightly improved in the absence of RSV. In groups of 1 Gy irradiation in the absence of RSV, the sperm count was slightly lower, and the proportion of malformed spermatozoa was importantly higher collated to results of 1 Gy separately. The germ cells parameters during the convalescence in the attendance of RSV were a little better, especially after irradiation with 0.5 Gy.

Results of convalescence showed that mice exhausted by 2 weeks irradiation respond poorly to further supplementation of RSV and the effect of such administration is adverse and may reflect the toxic long-lasting effect of RSV, which was so far not exactly recognized.

In the case of convalescence, if supplementation of RSV was started since the eighth day after the first irradiation, the presence of resveratrol, especially after the combination of higher doses, increased the percentage of abnormal spermatozoa. Additionally, there was a decrease in the epididymis weight and the sperm count if RSV was combined with 1 Gy of irradiation and after 0.5 Gy + 7 mg/kg bw RSV. In the absence of RSV, the sperm count was reduced compared to results of irradiation separately and the frequency of malformed spermatozoa was markedly increased except for the combination 1 Gy + 8 mg/kg bw RSV, where the percentage of abnormal spermatozoa was similar. The only advantage is that combination of 1 Gy with each dose of RSV reduces the level of DNA fragmentation. The present study regarding male germ cells confirmed the earlier results for somatic cells where supplementation of RSV after the end of irradiation delayed the convalescence [28].

Previous studies have shown that the diminished sperm count following irradiation is dominantly assigned to germ cell apoptosis, with genetic aberrations probably mediated by Tp53 [8]. This is further supported by findings indicating that decreased sperm count coincides with degenerating seminiferous tubules, vacuolations, and mitochondrial defects in germ cells, suggesting the initiation of apoptosis [97]. Conversely, RSV has been reported to mitigate toxic effects, such as disrupted seminiferous tubules with few sperms in their lumens, abnormal sperm morphology, and vacuolations induced by atrazine [108], and to improve the histological integrity of testes in mice exposed to lead [109]. It has also been demonstrated that RSV reduces apoptotic germ cell counts and improves epithelial cell height in seminiferous tubules under conditions of chemically induced testicular toxicity, while restoring serum testosterone, LH, and FSH levels []. Furthermore, RSV mitigates testicular toxicity induced by immune checkpoint inhibition, primarily through activation of the nuclear factor erythroid 2-related factor 2 (NRF2) pathway and restoration of the solute carrier family 7 member 11 (SLC7A11)–glutathione (GSH)–glutathione peroxidase 4 (GPX4) axis. These effects include reduction in oxidative stress and ferroptosis (an iron-dependent cell death), improvement of blood–testis barrier integrity, normalization of gonadal hormone levels, and attenuation of inflammatory infiltration in testicular tissue [110]. The investigation of histological effects of co-administration of irradiation and RSV would be interesting.

Outcomes obtained are very important and promising in the point of view of public and reproductive health. They are consistent with the “One Health” concept.

There are known models for adaptative and radioprotective responses at cellular and system levels (e.g., stochastic dose-response models, biphasic adaptive kinetics, and feedback redox regulation). With suitable modification, such modeling approaches might be extended to describe time-dependent effects of post-irradiation interventions like RSV supplementation to enrich the mechanistic depth of the study and provide a theoretical link between empirical data and predictive biology.

## 5. Conclusions

Based on the results, we concluded that resveratrol is a very useful compound to improve the sperm count diminished by irradiation. RSV counteracted the killing of germ cells by ionizing radiation both after the start of RSV administration since 24 h as well as since the eighth day following the irradiation. In the occurrence of combination of high doses of irradiation with RSV since 24 h the mitigation of DNA damage was also observed. RSV may work as both radioprotector and radiomitigator of lethal effects in male gametes. Contrarily, supplementation during recovery after irradiation is not recommended since it may be toxic during long-lasting exposure, thus worsening the condition of the body and gametes.

## Figures and Tables

**Table 1:** The effects of resveratrol supplementation since 24 h after the start of irradiation on the sperm count and quality.

Daily Dose	Mean Body Weight (g) ± SD	Mean Testes Weight (mg) ± SD	Mean Epididymis Weight (g) ± SD	Sperm Count × 10^6^/mL ± SD	Percent of Motile Spermatozoa ± SD	Percent of Abnormal Spermatozoa ± SD	Comet Tail Moment ± SD	Percent of DNA in Comet Tail ± SD
Control	37.79 ± 2.93	258.90 ± 33.58	244.00 ± 37.32	6.23 ± 0.99	35.93 ± 17.80	17.36 ± 4.49	3.60 ± 3.41	8.17 ± 5.20
0.5 Gy	34.83 ± 4.17	192.70 ± 31.87 *	159.80 ± 34.34 *	4.05 ± 1.33 *	14.95 ± 7.35 *	19.63 ± 6.23	3.87 ± 2.75	8.06 ± 4.34
1 Gy	30.59 ± 5.17 *	208.00 ± 46.10 *	178.30 ± 54.28 *	2.88 ± 1.47 *	12.08 ± 7.21 *	22.08 ± 6.88	4.01 ± 2.42	8.47 ± 3.94
7 mg/kg RSV	32.96 ± 4.36 *	235.33 ± 48.29	198.33 ± 51.10	5.42 ± 4.40	24.82 ± 17.92	19.51 ± 6,96	4.97 ± 2.95	10.41 ± 4.70
28 mg/kg RSV	34.03 ± 2.41	215.13 ± 59.86	217.50 ± 22.97	4.71 ± 1.61	16.36 ± 7.18 *	22.54 ± 8.06	5.79 ± 2.66	11.82 ± 4.02
0.5 Gy + 7 mg/kg RSV	28.65 ± 2.54 *^a^	172.39 ± 28.15 *	231.88 ± 75.90 ^a^	3.98 ± 1.79	9.94 ± 16.26 *	23.69 ± 7.99 *	7.58 ± 5.10 ^a^	12.32 ± 4.27 *^a^
0.5 Gy + 28 mg/kg RSV	29.76 ± 3.73 *^a^	173.75 ± 25.09 *	227.00 ± 60.74 ^a^	4.47 ± 1.75	16.44 ± 13.43 *	19.69 ± 5.80	3.33 ± 2.85	6.03 ± 2.75
1 Gy + 7 mg/kg RSV	29.01 ± 3.73 *	170.57 ± 13.54 *^b^	188.57 ± 36.13 *	4.66 ± 2.08 ^b^	19.21 ± 11.18 *	19.27 ± 3.64	2.07 ± 1.37	5.07 ± 1.50
1 Gy + 28 mg/kg RSV	27.14 ± 3.66 *	162.57 ± 20.31 *^b^	191.57 ± 64.21 *	4.62 ± 2.15 ^b^	13.71 ± 7.71 *	19.59 ± 7.30	1.80 ± 1.59 ^b^	4.36 ± 2.81 ^b^

* *p* < 0.05 compared to control; ^a^ *p* < 0.05 compared to 0.5 Gy alone; ^b^ *p* < 0.05 compared to 1 Gy alone by post hoc Fisher’s test.

**Table 2:** The effects of resveratrol supplementation since eighth day after the start of irradiation on the sperm count and quality.

Daily Dose	Mean Body Weight (g) ± SD	Mean Testes Weight (mg) ± SD	Mean Epididymis Weight (g) ± SD	Sperm Count × 10^6^/mL ± SD	Percent of Motile Spermatozoa ± SD	Percent of Abnormal Spermatozoa ± SD	Comet Tail Moment ± SD	Percent of DNA in Comet Tail ± SD
Control	37.79 ± 2.93	260.50 ± 31.52	248.33 ± 36.82	6.22 ± 1.00	35.93 ± 17.80	20.18 ± 3.29	3.89 ± 2.53	8.27 ± 3.66
0.5 Gy	34.83 ± 4.17	192.70 ± 31.87 *	159.80 ± 34.34 *	4.05 ± 1.33 *	14.95 ± 7.35 *	22.40 ± 6.11	3.87 ± 2.75	8.06 ± 4.34
1 Gy	30.59 ± 5.17 *	204.10 ± 38.47 *	183.40 ± 63.66 *	3.01 ± 1.68 *	12.08 ± 7.21 *	21.93 ± 6.66	4.01 ± 2.42	8.47 ± 3.94
7 mg/kg RSV	32.90 ± 3.80	218.60 ± 30.12	226.40 ± 71.90	5.98 ± 2.05	9.30 ± 7.45 *	17.38 ± 4.03	5.71 ± 3.00	11.26 ± 4.33
28 mg/kg RSV	34.86 ± 2.36	245.80 ± 34.12	197.20 ± 38.28	5.83 ± 1.56	8.30 ± 7.80	19.64 ± 5.31	5.72 ± 1.30	11.16 ± 1.36
0.5 Gy + 7 mg/kg RSV	33.22 ± 2.32	213.00 ± 42.22 *	253.33 ± 41.01	4.49 ± 2.07	10.59 ± 7.85 *	20.80 ± 8.90	3.85 ± 2.08	7.96 ± 3.70
0.5 Gy + 28 mg/kg RSV	33.20 ± 2.80 *	218.33 ± 13.22 *	191.33 ± 30.53 *	6.25 ± 1.91	16.94 ± 11.32	16.18 ± 4.25	3.91 ± 1.68	7.91 ± 2.94
1 Gy + 7 mg/kg LYC	27.23 ± 2.62 *	183.86 ± 27.69 *	255.57 ± 44.38 ^b^	3.63 ± 1.64 *	8.72 ± 5.94 *	18.08 ± 6.58	4.25 ± 1.66	8.40 ± 2.44
1 Gy + 28 mg/kg LYC	28.60 ± 3.04 *	186.29 ± 49.88 *	227.71 ± 44.66	3.96 ± 2.30 *	12.71 ± 12.55 *	23.60 ± 7.45	6.03 ± 4.78 ^b^	11.26 ± 7.31

* *p* < 0.05 compared to control; ^b^ *p* < 0.05 compared to 1 Gy alone by post hoc Fisher’s test.

**Table 3:** The effects of resveratrol supplementation since 24 h after the start of irradiation on the sperm count and quality–effects after convalescence.

Daily Dose	Mean Body Weight (g) ± SD	Mean Testes Weight (mg) ± SD	Mean Epididymis Weight (g) ± SD	Sperm Count × 10^6^/mL ± SD	Percent of Motile Spermatozoa ± SD	Percent of Abnormal Spermatozoa ± SD	Comet Tail Moment ± SD	Percent of DNA in Comet Tail ± SD
Control	36.72 ± 3.94	246.06 ± 91.44	145.60 ± 91.66	6.39 ± 2.31	18.09 ± 16.73	21.98 ± 4.66	2.60 ± 0.99	6.24 ± 1.91
0.5 Gy	39.06 ± 4.17	158.00 ± 83.02 *	223.11 ± 64.49	3.09 ± 2.07 *	20.17 ± 15.61	25.66 ± 14.15	2.26 ± 0.60	5.73 ± 1.44
1 Gy	33.97 ± 7.55	216.00 ± 128.92	245.70 ± 107.4 *	3.14 ± 2.04 *	34.94 ± 17.83 *	26.44 ± 11.40	2.32 ± 0.66	5.96 ± 1.41
7 mg/kg RSV	39.24 ± 3.47	236.38 ± 36.98	212.44 ± 123.45	6.04 ± 2.10	20.13 ± 17.52	20.21 ± 5,65	2.85 ± 1.93	6.59 ± 3.65
28 mg/kg RSV	36.47 ± 2.41	221.89 ± 574.29	192.10 ± 34.38	6.14 ± 2.18	16.46 ± 7.56	19.20 ± 2.70	3.32 ± 2.06	7.05 ± 4.11
0.5 Gy + 7 mg/kg RSV RPR	32.50 ± 3.30 *^a^	128.50 ± 40.37 *^a^	147.00 ± 38.68	2.45 ± 1.19 *	8.34 ± 8.34 ^a^	34.91 ± 19.94 *	2.38 ± 1.18	5.74 ± 2.18
0.5 Gy + 28 mg/kg RSV RPR	33.77 ± 3.70 *^a^	91.80 ± 37.97 *^a^	213.20 ± 57.36	2.58 ± 1.12 *	14.23 ± 12.60	36.35 ± 15.32 ^a^	2.70 ± 1.76	5.65 ± 2.74
0.5 Gy + 7 mg/kg RSV RAR	27.49 ± 6.57 *^a^	160.96 ± 33.56 *	184.00 ± 42.61	3.60 ± 1.43	9.71 ± 10.94	29.98 ± 10.85	2.58 ± 0.83	4.36 ± 1.99
0.5 Gy + 28 mg/kg RSV RAR	29.49 ± 2.86 *^a^	179.27 ± 18.41 *	238.36 ± 46.91 *	3.34 ± 2.72	17.86 ± 11.47	26.22 ± 16.55	2.83 ± 1.75	5.21 ± 1.18
1 Gy + 7 mg/kg RSV RAR	28.60 ± 6.47 *^b^	176.87 ± 21.55 *	177.00 ± 66.73	2.42 ± 0.93 *	15.75 ± 9.29	40.20 ± 35.20 *	1.86 ± 1.60	4.03 ± 3.18
1 Gy + 28 mg/kg RSV RAR	26.40 ± 4.06 *	170.75 ± 16.48 *	224.33 ± 49.35	2.83 ± 0.48 *	14.58 ± 11.29	46.70 ± 15.14 *^b^	3.54 ± 0.85	6.77 ± 1.38

* *p* < 0.05 compared to control; ^a^ *p* < 0.05 compared to 0.5 Gy alone; ^b^ *p* < 0.05 compared to 1 Gy alone by post hoc Fisher’s test.

**Table 4:** The effects of resveratrol supplementation since eighth day after the start of irradiation on the sperm count and quality–after convalescence.

Daily Dose	Mean Body Weight (g) ± SD	Mean Testes Weight (mg) ± SD	Mean Epididymis Weight (g) ± SD	Sperm Count × 10^6^/mL ± SD	Percent of Motile Spermatozoa ± SD	Percent of Abnormal Spermatozoa ± SD	Comet Tail Moment ± SD	Percent of DNA in Comet Tail ± SD
Control	37.14 ± 2.72	263.42 ± 60.42	139.60 ± 35.08	6.19 ± 1.09	16.72 ± 9.80	18.56 ± 4.26	3.34 ± 1.05	7.48 ± 1.61
0.5 Gy	38.72 ± 4.32	176.00 ± 90.95 *	189.50- ± 41.44	3.35 ± 2.49 *	24.70 ± 11.62	29.13 ± 9.13	2.11 ± 2.75	4.64 ± 1.87
1 Gy	33.07 ± 4.10	186.50 ± 115.18 *	185.57 ± 96.55	4.97 ± 2.65	9.48 ± 5.22	33.88 ± 23.57 *	2.92 ± 2.16	6.60 ± 3.26
7 mg/kg RSV	35.14 ± 2.62	240.63 ± 85.54	120.71 ± 49.42	5.36 ± 2.38	9.46 ± 8.49	16.63 ± 3.05	4.60 ± 2.09	9.77 ± 3.92
28 mg/kg RSV	35.36 ± 2.72	234.71 ± 107.54	120.29 ± 58.22	5.96 ± 2.50	13.55 ± 8.19	18.89 ± 5.06	3.64 ± 1.53	7.61 ± 2.05
0.5 Gy + 7 mg/kg RSV RPR	32.21 ± 4.89 *	87.67 ± 11.81 *^,a^	155.67 ± 58.07	3.67 ± 1.77 *	18.11 ± 10.77	34.52 ± 13.15 *	3.28 ± 1.63	5.94 ± 3.10
0.5 Gy + 28 mg/kg RSV RPR	33.24 ± 2.80 *	99.75 ± 27.26 *^,a^	187.17 ± 58.07	2.81 ± 0.95 *^,a^	19.32 ± 8.85	35.57 ± 8.72 *	3.14 ± 2.30	6.75 ± 4.21
1 Gy + 28 mg/kg RSV RPR	32.33 ± 3.26 *	82.75 ± 15.78 *^,b^	100.50 ± 57.24 ^b^	1.67 ± 1.24 *^,b^	10.81 ± 3.9	46.95 ± 16.55 *^,b^	3.50 ± 2.23	8.81 ± 6.38
0.5 Gy + 7 mg/kg RSV RAR	31.60 ± 2.26 *^,a^	79.33 ± 12.50 *^,a^	165.33 ± 68.41	2.60 ± 2.66 *	13.75 ± 13.28	37.82 ± 16.41 *	1.76 ± 1.78	7.21 ± 3.21
0.5 Gy + 28 mg/kg RSV RAR	33.20 ± 3.80 *^,a^	218.57 ± 19.71	229.14 ± 52.55 *	2.34 ± 1.33 *	15.07 ± 11.26	37.3 ± 12.23 *	2.71 ± 3.29	4.37 ± 2.88
1 Gy + 7 mg/kg RSV RAR	28.87 ± 2.12 *	212.40 ± 15.57	237.2 ± 39.19 *	2.45 ± 1.57 *^,b^	8.57 ± 7.87 *	40.9 ± 6.21 *	2.74 ± 0.83	2.17 ± 0.39 *
1 Gy + 28 mg/kg RSV RAR	28.20 ± 3.46 *	203.0 ± 23.07	257.33 ± 38.73 *	1.77 ± 1.55 *^,b^	24.13 ± 16.40 ^b^	32.04 ± 13.87 *	3.02 ± 0.96	2.27 ± 1.84 *

* *p* < 0.05 compared to control; ^a^ *p* < 0.05 compared to 0.5 Gy alone; ^b^ *p* < 0.05 compared to 1 Gy alone by post hoc Fisher’s test.

## Data Availability

The original contributions presented in this study are included in the article. Further inquiries can be directed to the corresponding author.
